# Development and Evaluation of an Algorithm for the Computer-Assisted Segmentation of the Human Hypothalamus on 7-Tesla Magnetic Resonance Images

**DOI:** 10.1371/journal.pone.0066394

**Published:** 2013-07-23

**Authors:** Stephanie Schindler, Peter Schönknecht, Laura Schmidt, Alfred Anwander, Maria Strauß, Robert Trampel, Pierre-Louis Bazin, Harald E. Möller, Ulrich Hegerl, Robert Turner, Stefan Geyer

**Affiliations:** 1 Department of Psychiatry and Psychotherapy, University Hospital Leipzig, Leipzig, Germany; 2 Department of Neuropsychology, Max Planck Institute for Human Cognitive and Brain Sciences, Leipzig, Germany; 3 Department of Neurophysics, Max Planck Institute for Human Cognitive and Brain Sciences, Leipzig, Germany; 4 Nuclear Magnetic Resonance Unit, Max Planck Institute for Human Cognitive and Brain Sciences, Leipzig, Germany; University of Maryland, United States of America

## Abstract

*Post mortem* studies have shown volume changes of the hypothalamus in psychiatric patients. With 7T magnetic resonance imaging this effect can now be investigated *in vivo* in detail. To benefit from the sub-millimeter resolution requires an improved segmentation procedure. The traditional anatomical landmarks of the hypothalamus were refined using 7T T1-weighted magnetic resonance images. A detailed segmentation algorithm (unilateral hypothalamus) was developed for colour-coded, histogram-matched images, and evaluated in a sample of 10 subjects. Test-retest and inter-rater reliabilities were estimated in terms of intraclass-correlation coefficients (ICC) and Dice's coefficient (DC). The computer-assisted segmentation algorithm ensured test-retest reliabilities of ICC≥.97 (DC≥96.8) and inter-rater reliabilities of ICC≥.94 (DC = 95.2). There were no significant volume differences between the segmentation runs, raters, and hemispheres. The estimated volumes of the hypothalamus lie within the range of previous histological and neuroimaging results. We present a computer-assisted algorithm for the manual segmentation of the human hypothalamus using T1-weighted 7T magnetic resonance imaging. Providing very high test-retest and inter-rater reliabilities, it outperforms former procedures established at 1.5T and 3T magnetic resonance images and thus can serve as a gold standard for future automated procedures.

## Introduction

### Background

The hypothalamus is a small grey matter brain region of the diencephalon that surrounds the anterior portion of the third ventricle. As a part of the limbic system it connects the cerebral cortex with the visceral system and hence is deemed a ‘mediator between the mind and the body’ – both affected in psychiatric diseases. Structural correlates have been described for example with mood disorders [1], [2], schizophrenia [3], [4], anxiety [5], borderline personality disorder [6], narcolepsy [7], [8], and frontotemporal dementia [9]. Since the hypothalamic region is less than 4 cm^3^ in size, and hard to distinguish from its surroundings, sub-millimeter magnetic resonance imaging (MRI) is essential for its *in vivo* structural investigation. But to benefit from the improved resolution a high measurement accuracy of the volumetric technique is vitally important. To this end, (semi-) automated segmentation algorithms might seem preferable, but existing software applications are limited to segmenting tissue types or larger brain structures. In case of the hypothalamus the tissue segmentation approach is bound to fail at least superiorly and laterally, where the hypothalamic grey matter is bordered by non-hypothalamic grey structures [10]. Therefore manual segmentation guided by anatomical landmarks is still state-of-the-art for defining the human hypothalamus on MR images.

### Macrostructural boundaries of the hypothalamus in humans

Disagreement exists regarding the actual size of the hypothalamus in healthy humans. Whereas an early and often-cited histological study by Stephan et al. [11] estimated the human hypothalamus to be 3.6 cm^3^ in volume, recent histological or neuroimaging studies reported it to be no more than half this size (Table 1). The variability of the reported volumes might be caused by different methodological approaches. As an exceptional example, Stephan and his colleagues projected a sample of Cresyl-violet-stained brain sections on photographic paper, which was then cut and weighted. Based on the weight of the photographic paper representing the structure of interest, the volume was estimated, corrected for tissue shrinkage and corrected again for the difference between the individual's brain weight and the standard brain weight of humans. In contrast, researchers can now interactively delineate brain structures on the computer monitor that presents the full head of a living subject in 3D view. Given that the used MR sequence is not sensitive to distortions or other imaging artifacts the volume can be determined without the need for correction and conversion factors. The delineated volume is directly accessible as the number of marked voxels (volume image elements).

**Table 1:** Volumetric estimations of the hypothalamus in humans.

	Volumetric method	Region of interest	Volume (cm^3^ * ± SD)	Test-retest reliability	Inter-rater reliability
Bielau et al. [1]	*Post mortem* stereology	Hypothalamus bilateral	22 C: 1.41±0.30	Intercorrelation coefficient = .89 (N = 10)	Intercorrelation coefficient = .85 (N = 10)
Bogerts [38] †	*Post mortem* stereology	Hypothalamus unilateral	14 women: 0.66±0.10; 9 men: 0.77±0.10	n/a	n/a
Callen et al. [15]	1.5T MRI *in vivo*	Hypothalamus uni-lateral (MB excluded)	40 C: 0.30±0.04	ICC>.90 (N = 20)	ICC>.87 (N = 20)
Goldstein et al. [3]	1.5T MRI *in vivo*	Hypothalamus bilateral (fornices included)	21 women: 0.78±0.16; 27 men: 0.92±0.11	n/a	ICC = .81 (N = 10)
Hulshoff Pol et al. [19]	1.5T MRI *in vivo*	Hypothalamus bilateral (MBs excluded; scan 1)	6 women: 1.00±0.05; 9 men: 1.05±0.18	ICC = .86 (N = 10)	n/a
Klomp et al. [18] ‡	1.5T MRI *in vivo*	Hypothalamus bilateral (MB excluded)	156 C: 1.04±0.14	ICC = .96 (N = 10)	n/a
Koolschijn et al. [17] ‡	1.5T MRI *in vivo*	Hypothalamus bilateral (MBs excluded)	11 C1MZ: 1.04±0.10; 11 C2MZ: 1.01±0.14; 11 C1DZ: 0.97±0.13; 11 C2DZ: 1.04±0.13	ICC = .91 (N = 10)	ICC = .93 (N = 10)
Makris et al. [16] #	1.5T MRI *in vivo*	Hypothalamus bilateral (fornices included)	18 women: 0.79±14; 26 men: 0.91±0.11	n/a	see Goldstein et al. [3]
Peper et al. [20] ‡	1.5T MRI *in vivo*	Hypothalamus bilateral	36 boys: 1.05±0.12; 40 girls: 1.01±0.09	ICC = .86 (N = 10)	n/a
Piquet et al. [9]	3T MRI *in vivo* and *post mortem* stereology	Anterior and posterior hypothalamus unilateral	Stereology: 6 C: 0.16±0.04 (anterior); 0.19±0.04 (posterior)	Stereology: ICC = .995 (N = 3; 5 repetitions); MRI: ICC = .964 (N = 5)	n/a
Stephan et al. [11]	*Post mortem* stereology	Hypothalamus bilateral	21 homo sapiens sapiens: 3.56	n/a	n/a
Terlevic et al. [5] ‡	1.5T MRI *in vivo*	Hypothalamus right and left (MB excluded, fornix included)	21 C: 0.36±0.04 (right); 0.34±0.03 (left)	n/a	ICC>.9 (N = 20)
Tognin et al. [4] #	3T MRI *in vivo*	Hypothalamus right and left (MB excluded, fornix included)	26 C: 0.36±0.05 (right); 0.36±0.04 (left)	ICC>.90 (N = 10)	ICC>.90 (N = 20)

C: control; DZ: dizygotic twin; ICC: intraclass correlation coefficient; MB: mamillary body; MRI: magnetic resonance imaging; MZ: monozygotic twin; N: sample size; n/a: not applicable;

* for clarity, millilitres or cubic millimetres are converted into cubic centimetres;

† Segmentation procedure and sample from Bielau et al. [1] plus 1C;

‡ segmentation procedure adapted from Hulshoff Pol et al. [19];

# segmentation procedure and subsample from Goldstein et al. [3].

Another important source of variance can be found in the different definitions of the hypothalamic region. In the strict sense, the anterior hypothalamus begins at a coronal plane that intersects the foramen of Monro and the middle of the optic chiasm [12]. However, since the telencephalic preoptic area is extensively intertwined with the diencephalic hypothalamus, both regions are usually treated together. The anterior border of the hypothalamus (including the preoptic region) is thus generally considered to be the lamina terminalis [12]–[14] which is barely visible on standard MR images but coincides with the prominent anterior commissure. Most imaging studies started the segmentation of the hypothalamus on the first slice on which the anterior commissure appears continuous [3]–[5], [15]–[17]. In many brains this should also be the ‘first slice on which the optic tract is attached to the brain by two “wisps” of white matter’ [9]. If the anterior margin is set at the first slice posterior to the continuous anterior commissure [18]–[20] a substantial portion of the preoptic hypothalamus might be excluded [21].

For the sake of repeatability, the posterior end of the hypothalamus was usually set at the posterior end of the mamillary bodies [1], [3], [9], [13], [20]. This definition tolerates a potential loss of the posterior tips of the lateral hypothalamic area and posterior hypothalamic area which can extend beyond the mamillary bodies [12], [22], [23]. Sometimes, the mamillary region was excluded from the hypothalamus by stopping in front of the mamillary bodies, or with the appearance of the fornices [4], [5], [15], [17].

Defining the superior boundary of the hypothalamus is challenging because it adjoins the ventral thalamus which has similar intensity in T1-weighted images [10], [24]. Several imaging studies chose the imaginary line between the anterior and posterior commissures (AC-PC) as the uppermost boundary for the hypothalamus [13], [17]–[19], [25]. However, since the superoinferior position of the anterior commissure can vary from brain to brain, using the AC-PC throughout the entire hypothalamus risks losing a portion of the hypothalamus [14]. Alternatively, the hypothalamic sulcus can serve as major superior boundary of the hypothalamus posterior to the anterior commissure [5], [9], [12], [16]. This is a suitable landmark for most substructures of the hypothalamus, with the exception of the paraventricular nucleus, which can extend beyond this line [14], [26]. In addition, the superior end of the fornix [15], the posterior limb of the internal capsule, and the mamillothalamic tract [3], [4], [16] can be used as landmarks.

The hypothalamus constitutes the floor of the diencephalon. Anteriorly it rests atop the optic chiasm and is then encircled by the posteriorly emerging optic tracts – both reliable landmarks in MR images [24]. Immediately posterior to the optic chiasm, the hypothalamic tuber cinereum forms the infundibular recess, from which projects the hypophyseal stalk that contains the median eminence. Although the infundibular (or arcuate) nucleus of the hypothalamus extends into the median eminence [14], [27], the hypophyseal stalk was generally defined as inferior border of the human hypothalamus on MR images [3], [16]–[19].

Surrounded anteriorly and inferiorly by the (bilateral) hypothalamus, the third ventricle is such a prominent landmark that few segmentation procedures made the effort to define any other medial boundary. For unilateral segmentation the interhemispheric fissure can be used to separate the right from the left hypothalamus [3], [16].

Due to the lack of distinct boundaries in this region, the lateral edge of the hypothalamus certainly poses the greatest challenge for MRI-based segmentation of the hypothalamus. As an approximation a straight line can be drawn from the lateral edge of the optic tract to the fornix, to the internal capsule, or to the hypothalamic sulcus [9], [15]. This definition might suffice for some purposes, but at least in the mamillary region of the hypothalamus the lateral edge of the optic tract is a questionable landmark [23]. An alternative approach identifies the internal capsule, “vicinity of the globus pallidus”, and cerebral peduncle based on their higher intensities on standard MR images [16], [24], [28]. Accordingly, a suitable segmentation of the hypothalamus would be achieved by defining all low intensity voxels up to the lateral “white matter” as hypothalamic [17]–[20]. From this region the low intensity substantia nigra should also be excluded [3] as well as the subthalamic nucleus [12], [23], [29]. In addition, *post mortem* investigations revealed a diffuse transition area between the preoptic hypothalamic region and the brighter internal capsule or dark globus pallidus at its side [1], [23]. In this region the lateral hypothalamic area adjoins non-hypothalamic grey matter structures, for example the substantia innominata (including the basal nucleus of Meynert), the great terminal island, and the bed nucleus of the stria terminalis [12], [23]). Difficult to detect with the naked eye on MR images, this transition area should show slightly higher intensities than the hypothalamus, which is caused by intermingled white matter fibres such as the diagonal band of Broca, the medial forebrain bundle, the sublenticular stria, or the ventral amygdalofugal pathway [23]. In approximation, the lateral edge of the optic tract might be used as a landmark for the lateral border of the preoptic hypothalamus [3], [4].

As reviewed above, several of the macroscopic landmarks used for the MRI-based segmentation of the hypothalamus are arbitrary. With the exception of the procedure described by Piquet et al. [9], they were developed on 1.5T MR images, and many of them prove approximate when employed on 7T images. Moreover, almost all existing segmentation procedures restricted the segmentation to the coronal plane, ignoring vital information that could be gained from a triplanar view. To utilise the high resolution of 7T MRI, a refinement of the landmarks and segmentation procedure was necessary. In due consideration of the previous works, and with reference to the coronal microanatomical atlas from Mai et al. [23], we have developed a detailed segmentation algorithm for the hypothalamus on 7T MR images. With the help of the triplanar myeloarchitectural atlas from Riley [22], the landmarks were defined not only for the coronal view, but also for the sagittal and transverse planes. To optimise reliability and reduce working time, the segmentation algorithm was then adapted to MR images in a colour-coded format. In terms of reproducibility, benchmarks set previously by histological or neuroimaging studies needed to be met (Table 1). For our segmentation procedure to be reproduced by other researchers, inter-rater reliability was of particular interest. In the past, schematic approximations for the superior and lateral hypothalamic boundaries helped to ensure intraclass correlation coefficients (ICC) of up to ICC = .93 [15], [17]. More precise segmentation procedures still achieved inter-rater reliabilities of at least ICC = .81 [3], [4].

## Methods

### Image acquisition and preprocessing

Data from 10 subjects (8 female; age 38.5±13.6 years) without history of neurological diseases were analysed for the evaluation of the segmentation algorithm. All subjects had given written informed consent and the study was approved by the Ethics Committee of the University of Leipzig.

Whole brain T1-weighted images were acquired with a 7T whole-body MR scanner (MAGNETOM 7T, Siemens, Erlangen, Germany) and a 24-channel NOVA head coil (Nova Medical, Inc., Wilmington MA, USA). A 3D magnetisation-prepared 2 rapid acquisition gradient echoes sequence (3D MP2RAGE [30]) was used with repetition time (TR) = 8250 ms; inversion times (TI1/TI2) = 1000/3300 ms; flip angles (FA1/FA2) = 7°/5°; echo time (TE) = 2.51 ms; bandwidth (BW) = 240 Hz/Px, 1 average. A field of view (FOV) of 224×224 mm^2^; an imaging matrix of 320×320; and 240 slices with a thickness of 0.7 mm resulted in a nominal acquisition voxel size of 0.7 mm isotropic. By accelerating the acquisition using parallel imaging (GRAPPA [31]; acceleration factor = 2), a scan time of 18:02 min was achieved.

The uniform MP2RAGE images, referred to as T1-weighted images in the rest of this text, were skull-stripped using Medical Image Processing and Visualization (MIPAV [32]) software (version 5.3.4). The images were then co-registered into a coordinate system compatible to the atlas by Mai et al. [23], which differs from Talairach space [33] with respect to the intercommissural line that connects the *centres* of the anterior and posterior commissures. The registered images were then interpolated to an isotropic nominal voxel size of 0.5 mm using shifted linear interpolation [34]. The preprocessed greyscale images were cropped to an image matrix of 320×400×320 voxels covering the entire brain. The data was stored with 16-bit intensity (I) resolution but only the lower 12 data bit were used.

For MP2RAGE, image artifacts caused by distortions and point spread function-related blurring are expected to be negligible. Furthermore, the use of this particular sequence enables the acquisition of uniform T1-weighted images free from bias field effects [30] which are usually a problem at high field strengths such as 7T. However, strong bias field effects depending on shape and size of the subject's head can still result in local areas with non-perfect spin inversion, especially in the temporal lobes. To correct for the resulting differences in image brightness the intensity histograms of the 10 datasets were matched to another dataset using MIPAV. The reference dataset was not included in the volumetric analysis.

### Computer-assisted segmentation of the hypothalamus on 7T MR images

The hypothalamus segmentation was performed on 22″ computer screens using the freeware software application ITK-SNAP [35] version 2.1.4-rc1 for interactive masking in triplanar view.

**Figure 1:**
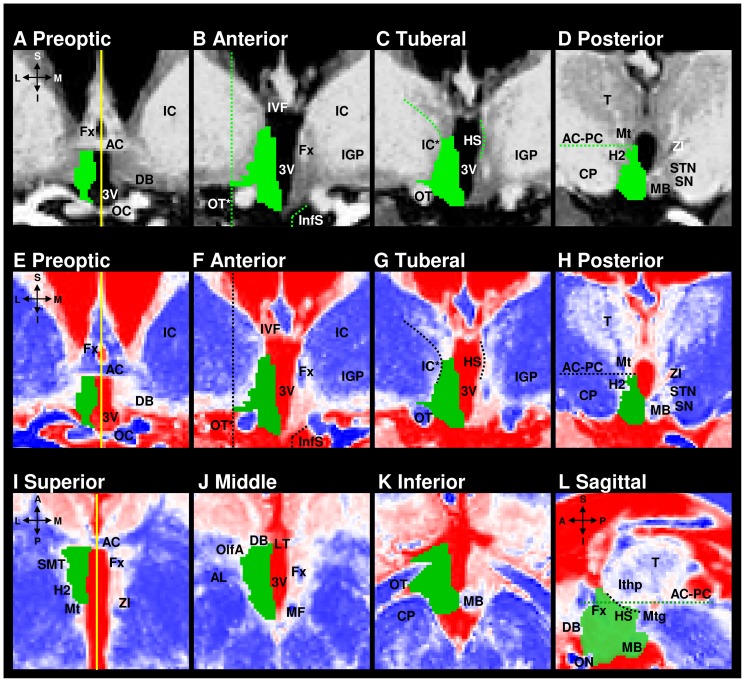
T1-weighted images with the hypothalamus masked unilaterally and corresponding anatomical landmarks. Coronal view (A–H, orientation identical to A), transverse view (I–K, orientation identical to I), and sagittal view (L). The yellow vertical lines in A, E, and I represent the midline of the brain. The left side of the images correspond to the right side of the brain. 3V: third ventricle, A: anterior, AC: anterior commissure, AC-PC (dotted line): imaginary line between anterior and posterior commissures, AL: ansa lenticularis, CP: cerebral peduncle, DB: diagonal band of Broca, Fx: column of the fornix, H2: lenticular fasciculus (field H2), HS (dotted line): hypothalamic sulcus, I: inferior, IC: internal capsule; IC* (dotted line): medial pole of internal capsule, IGP: internal globus pallidus, InfS (dotted line): junction with infundibular stalk, Ithp: inferior thalamic peduncle, IVF: interventricular foramen, L: lateral, LT: lamina terminalis, M: medial, MB: mamillary body, MF: mamillary fasciculus, Mt: mamillo-thalamic tract, Mtg: mamillo-tegmental tract, OC: optic chiasma, OlfA: olfactory area, ON: optic nerve, OT: optic tract, OT* (dotted line): vertical line through lateral edge of optic tract, P: posterior, S: sagittal, SMT: stria medullaris of thalamus, SN: substantia nigra, STN: subthalamic nucleus, T: Thalamus, ZI: zona incerta. The snapshots were taken with ITK-SNAP [35].

**Figure 2:**
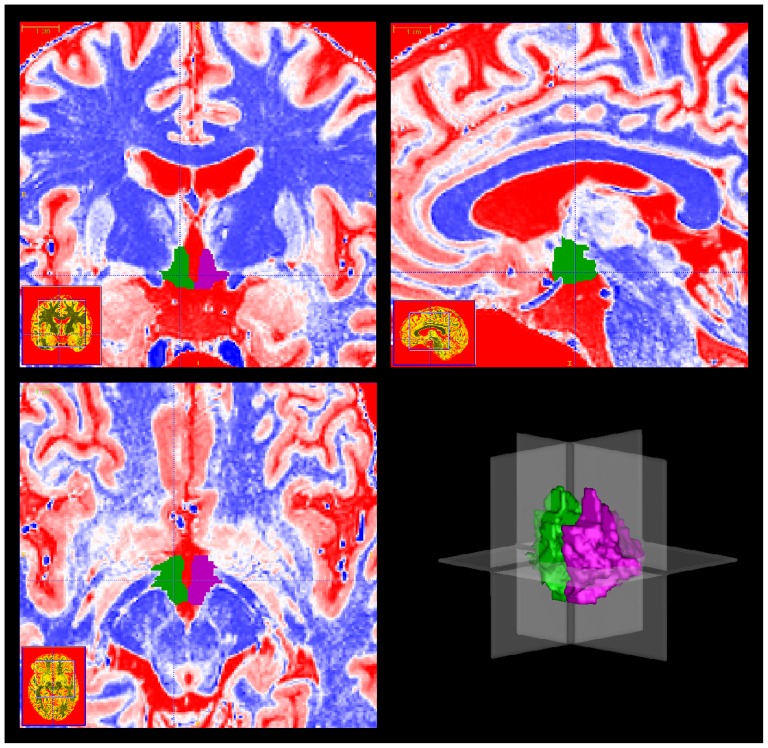
Triplanar view of the segmented left and right hypothalamus in colour-coded images. Coronal plane (top left), sagittal plane (top right), and transverse plane (bottom left) (cf. dotted lines in other planes). A 3D-reconstruction of the hypothalamus mask (both hemispheres, left anterolateral view) is shown in the lower right image. The left side of the images correspond to the right side of the brain. The crosshairs indicate the intersecting planes of the 3D-image. The snapshots were taken with ITK-SNAP [35].

The hypothalamic landmarks previously established on 1.5T and 3T MR images were refined with reference to the *post mortem* single brain atlases by Mai et al. [23] and Riley et al. [22]. Based on the new landmarks, a detailed algorithm for the computer-assisted manual segmentation of the hypothalamus on high-resolution T1-weighted 7T images was developed (Document S1, Figure 1). In short, the hypothalamus was segmented unilaterally in triplanar view with the help of macroanatomical landmarks and a colour-coding scheme that visually enhanced the borders between hypothalamic and non-hypothalamic structures (Figure 2). The region of interest (ROI) included the preoptic area, the column of the fornix posterior to the preoptic area, and the mamillary body. The optic tract, the mamillary fasciculus (bifurcating into mamillo-thalamic and mamillo-tegmental tract), the subthalamic nucleus, and the diffuse transition area lateral to the preoptic area, as described above, were excluded.

**Figure 3:**
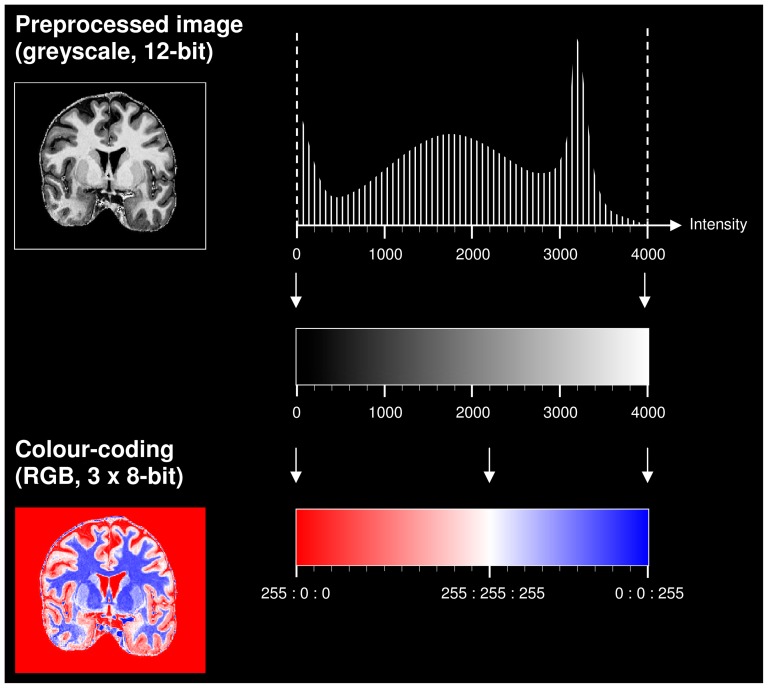
Schematic representation of the colour-coding procedure. The colour-coding of the preprocessed greyscale images (cf. histogram) was done by piecewise linear mapping from the input intensity (I) values in the range [0,4000] to the red-green-blue (RGB) colour space (3×8-bit). The colour red (255 ∶ 0 ∶ 0) was assigned to the intensity I = 0, white (255 ∶ 255 ∶ 255) to I = 2200, and blue (0 ∶ 0 ∶ 255) to I = 4000. The snapshots were taken with ITK-SNAP [35].

The segmentation algorithm was fully standardised. It defined the sequence of working steps and, in case of potentially conflicting landmarks, their hierarchical order – designated according to their anatomical validity. As a first step, the hypothalamus was divided into four anteroposterior compartments (preoptic, anterior, tuberal, posterior) in accordance with Goldstein et al. [3]. This was done on the greyscale images which had a fixed scale ranging from I = 0 (black) to I = 4000 (white, Figure 3). Secondly, the hypothalamus was delineated slice by slice in coronal plane following compartment-specific landmarks (Document S1; Figure 1, A–D). In a third step, the greyscale images were colour-coded by piecewise linear mapping from the greyscale intensity values in the range [0,4000] to the RGB colour space (3×8-bit). The colour red (255 ∶ 0 ∶ 0) was assigned to the intensity I = 0, white (255 ∶ 255 ∶ 255) to I = 2200, and blue (0 ∶ 0 ∶ 255) to I = 4000, as shown in Figure 3. The boundary voxels defined in the second step on the greyscale images could now be easily verified as indeed belonging to the hypothalamus (coded red or white) or to surrounding structures (coded blue), (Figure 1, E–L). The only structures coded blue to be included in the hypothalamus segmentation were the fornix and the pallidohypothalamic fibres running through the hypothalamus, and the mamillary body. The verification using the colour code was done in triplanar view to prevent implausible changes in the other canonical planes. It started (slice by slice) with the coronal plane, continued (slice by slice) with the transverse plane, and finished (slice by slice) with the sagittal plane. For the verification in the coronal plane the division of the hypothalamus into four anteroposterior compartments (and their respective landmarks) was kept. For the inspection in the transverse plane, the hypothalamus had to be subdivided further into three transverse levels, each corresponding to a distinct set of landmarks. Additional partitioning into mediolateral compartments was not needed because the sagittal landmarks were adequate throughout the entire mediolateral extent of the hypothalamus. The segmentation was completed after a process of re-checking.

Some anatomical terms of the segmentation algorithm (Document S1) may require explanation. First, in line with Mai et al. [23] and Nieuwenhuys et al. [12], the white matter lateral to the preoptic hypothalamus was identified as the diagonal band of Broca which merged superiorly with the medial forebrain bundle. The diagonal band was visible as a bright fibre bundle even with 1.5T MRI [24]. In contrast, Riley [22] observed the diagonal band (or “*fasciculus olfactorius*”) essentially anterior to the anterior commissure. He labelled the lateral vicinity of the preoptic hypothalamus “*area olfactoria*” which contained portions of the stria terminalis (“*stria semicircularis*”) and the “*stria medullaris thalami*”. Second, the stria medullaris of thalamus was observed by Mai et al. [23] only near the interventricular foramen, but in accordance to Riley [22] we identified it as an important lateral boundary of the hypothalamus in transverse view. Finally, the inferior thalamic peduncle of Mai et al. [23] corresponds to the *pedunculus ventralis thalami* of Riley [22].

After a short training period, two investigators traced the left and the right hypothalamus of the 10 subjects in independent segmentation runs. The segmentation runs were repeated by both raters after approximately two weeks.

### Statistics

Test-retest reliability of the computer-assisted segmentation algorithm was estimated in terms of ICC (model 1,1 of Shrout and Fleiss [36]) and the mean Dice's coefficient [37]. When multiplied by 100, Dice's coefficient represents the percentage of voxels overlapping in two images (percental voxel overlap), relative to the average number of voxels in the two images. The segmentation repetitions of each rater were tested for mean differences using paired t-tests (*p*<0.05 was considered significant).

Inter-rater reliability was calculated using ICC model 3,1 [36] and the mean Dice's coefficient. The volumes of the first segmentation runs of each rater were tested for mean differences between the two investigators and hemispheres using paired t-tests.

Normality of the morphometric data and the pair-differences were tested using the Kolmogorov-Smirnov test. The mean volume (mm^3^) of the left, right, and bilateral hypothalamus of the 10 subjects was calculated based on the voxels marked by rater 1 in the first segmentation run.

## Results

The tracing procedure took approximately 3 hours per ROI. The volumes and pair-differences between segmentation runs, hemispheres, and raters were normally distributed.

The test-retest reliabilities were high, with ICC (1,1) = .97 and higher. There were no significant mean differences between the segmentation repetitions of the investigators. The mean voxel overlap between the first and the second segmentation run was at least 96.8% for both investigators.

The inter-rater reliabilities were also high, with at least ICC (3,1) = .94. Of all voxels masked by one investigator during the first segmentation run, 95.2% were also segmented by the other rater (Table 2). Analyses of the first segmentation runs of the two investigators revealed no significant mean differences between two raters or hemispheres.

**Table 2:** Reliability of the computer-assisted segmentation.

	N = 10	Left hypothalamus	Right hypothalamus
Test-retest reliability			
	ICC (1,1)	≥.98	≥.97
	Dice's coefficient	≥96.8	≥97.3
Inter-rater reliability*			
	ICC (3,1)	.94	.97
	Dice's coefficient	95.2	95.2

* The first runs were assessed.

ICC: intraclass correlation coefficient.

Based on the voxels masked by the first rater during the first segmentation run, the mean volumes (± SD) of the hypothalamus were: Left: 554.56 mm^3^ (±50.47 mm^3^), right: 576.08 mm^3^ (±59.44 mm^3^), and bilateral: 1130.64 mm^3^ (±103.48 mm^3^). In the female subgroup the volumes were: Left: 536.39 mm^3^ (±37.16 mm^3^), right: 556.30 mm^3^ (±46.96 mm^3^), and bilateral hypothalamus: 1092.69 mm^3^ (±74.06 mm^3^).

## Discussion

We developed the first reproducible method for the volumetric measurement of the human hypothalamus with 7T MR images. Due to sub-millimeter resolution, we were able to refine the anatomical landmarks that have so far guided the segmentation of the hypothalamic region on MR images. Developed for a triplanar view, the segmentation algorithm exploits all structural data visible on the images. In addition, a colour-coded mode facilitates the identification of low-contrast boundaries between the anatomical landmarks and the hypothalamus. By adhering to the computer-assisted segmentation algorithm, high degrees of test-retest and inter-rater reliability were achieved. In fact, with nominal values of inter-rater reliability of ICC (3,1)≥.94 our segmentation algorithm outperforms existing methods established for 1.5T or 3T MRI.

Given the high variability of hypothalamus volumes reported in previous studies, it was expected that the volumes obtained by our method (approx. 1.1 cm^3^, bilateral hypothalamus) would lie within the range of previous estimations (approx. 0.6–3.6 cm^3^, see Table 1). More precisely, the volumes estimated in this study are larger than the numbers reported by histological and neuroimaging studies that employed schematic approximations [9], [15], [17]–[20]. This is to be expected, since these boundaries (e.g. AC-PC as superior border) are usually too conservative, as discussed above. In addition, most of these studies excluded the fornices running through the hypothalamus and the mamillary bodies. Compared with studies that segmented the hypothalamus *without* the help of approximations, our volume estimations lie between neuroimaging results (approx. 0.7–0.9 cm^3^ [3]–[5], [16]) and histological measurements (approx. 1.3–1.5 cm^3^ [1], [38]). Possible reasons for our conservative estimations compared to the volumes derived by *post mortem* histology [1], [38] might be found in the different methodical approaches. Whereas the *post mortem* measurements were based on Nissl-myelin staining, we used T1-weighted MR imaging, which is sensitive to a larger number of tissue properties like myelinisation, water content, and iron concentration. In addition, the *post mortem* volumes were corrected by the estimated shrinkage factor, which might have lead to overestimation of the actual hypothalamus volume. A more conservative definition of the hypothalamic region, used in our study, could also have contributed to the volume differences. We excluded the diffuse transition area lateral to the preoptic hypothalamus and the subthalamic nucleus to prevent our volume estimations to be affected by potentially non-hypothalamic structures.

A few important limitations of the computer-assisted segmentation algorithm must be considered. First, based on *in vivo* MRI data, our method cannot assess the microstructural (e.g. histological) validity of landmarks. A validation of our segmentation algorithm in *post-mortem* brains is therefore a mandatory task for the future. Histology is usually considered the gold standard for validation of borders between brain regions. But, as illustrated by the massive discrepancy between the early volume estimations by Stephan et al. [11] and recent studies, histology is also limited [39]. A comparison between a *post mortem* scan of the brain *in situ*, a second scan after fixation of the brain *ex situ*, and finally the histological analysis of the same brain might overcome these difficulties and provide new insights.

Second, histogram matching to an external reference was performed to eliminate differences in image brightness. As we used normalised images, such differences were minimal, mainly due to non-perfect spin inversion in areas with strong bias field. When adapting this protocol to other MRI data sets, a similar normalisation procedure and/or adaptation of the colour values of the voxels may become necessary.

Finally, the segmentation algorithm was developed for 7T MR images which facilitated the identification of anatomical boundaries. We expect that the algorithm performs similarly well on MR images with similar resolution acquired with lower field strength, but this remains to be proven in future studies.

Taken together, we present a computer-assisted algorithm for the manual segmentation of the human hypothalamus using T1-weighted images acquired at 7T. The hypothalamus is segmented separately for each hemisphere in triplanar view with the help of macroanatomical landmarks and colour coding that enhances the visibility of hypothalamic borders. With very high test-retest and inter-rater reliabilities, it outperforms earlier procedures developed for 1.5T and 3T MR images. This warrants that it can serve as a gold standard for the development and validation of future automated segmentation procedures. The estimated volumes lie within the range of previous measurements *ex vivo* (i.e., histology) and *in vivo* (i.e., neuroimaging). The reduction of the measurement error promises an improved exploitation of the high resolution of 7T MRI for hypothalamus volumetry. Our detailed segmentation procedure provides a good basis for landmark-based [25] or diffusion MRI-based [40] methods that map subdivisions of the hypothalamus *in vivo*. Once applied in larger samples of neuropsychiatric patients, high-resolution volumetry of the hypothalamus will improve our understanding of the pathogenesis of psychiatric disorders.

## Supporting Information

Document S1 **Segmentation guidelines for the human hypothalamus, unilateral.**
(DOC)
