# Supplementary material for: Development and Evaluation of an Algorithm for the Computer-Assisted Segmentation of the Human Hypothalamus on 7-Tesla Magnetic Resonance Images
Source: PLoS One. 2013 Jul 23;8(7):e66394. doi: 10.1371/journal.pone.0066394 (PMC3720799; doi:10.1371/journal.pone.0066394)
Supplement: Document S1 — Segmentation guidelines for the human hypothalamus, unilateral. (DOC) [file pone.0066394.s001.doc]

**Document S1. Segmentation guidelines for the human hypothalamus, unilateral.**

| **0. Segmentation conditions** | | | | | | |
| --- | --- | --- | --- | --- | --- | --- |
|  | Closed sunblind and ambient light. | | | | | |
|  | Standardised monitor settings (position, angle, brightness, contrast). | | | | | |
| **1. Anteroposterior compartmentalisation** | | | | | | |
|  | Triplanar view; greyscale images. | | | | | |
|  | Define midline and anteroposterior sections. | | | | | |
|  | If not stated otherwise, zoom to 1 voxel per 1.4 mm on monitor surface. | | | | | |
| **2. Coronal segmentation of hypothalamic grey matter** | | | | | | |
|  | Single plane view; coronal; greyscale images. | | | | | |
|  | Start at first slice with continuous anterior commissure and continue for each slice to the posterior end of the mamillary body. | | | | | |
|  | - | Define four boundary voxels, one for the superior border, one for the inferior border, one for the lateral border, and one for the medial border. | | | | |
|  | - | Connect the boundary voxels sequentially to create a continuous line of voxels (outline), using those voxels with maximum contrast to their neighbouring voxels. | | | | |
|  | - | Fill and fade the mask you have just created to check for any deviations from the underlying anatomical shape (change of zoom allowed). | | | | |
|  | - | Preferentially select landmarks in order of hierarchy – as described below (‘>’); in case of doubt select that which can be identified best. Landmarks divided by a comma (‘,’) are of the same hierarchical level. | | | | |
|  | **Coronal landmarks** | | **Inferior** | **Superior** | **Medial** | **Lateral** |
|  |  | **Preoptic hypothalamus** (first slice with ipsilateral continuous anterior commissure); (Figure 1A) | Optic chiasm | Anterior commissure, column of the fornix | Midline between hypothalami, third ventricle, lamina terminalis | Diagonal band > medial forebrain bundle > lateral edge of the optic chiasm |
|  |  | **Anterior hypothalamus** (first slice with ipsilateral inter-ventricular foramen ); (Figure 1B) | Junction* of optic tract and infundibular stalk | 1st slice: inferior to fornix, 2nd slice: lateral to fornix, 3rd slice: superior to fornix > medial pole of the internal capsule | Midline between hypothalami, third ventricle | Internal capsule, internal globus pallidus, max. lateral edge of the optic tract |
|  |  | **Tuberal hypothalamus** (anterior pole of ipsilateral anteroventral/ -medial thalamus); (Figure 1C) | Cerebral exterior | Hypothalamic sulcus > medial pole of the internal capsule > intersection of ventricle wall and extended superior edge of putamen | Midline between hypothalami, third ventricle | Internal capsule, internal globus pallidus |
|  |  | **Posterior hypothalamus** (anterior and posterior extent of ipsilateral MB); (Figure 1D) | Cerebral exterior | Hypoth. sulcus > inferior thalamic peduncle > medial pole of field H2 > mamillary fasciculus (incl. convergence with MB) > anterior commissure | Minimal intersection area* in sagittal plane between MBs | Zona incerta, cerebral peduncle, substantia nigra, subthalamic nucleus |

**Document S1. Continued**

| **3. Validation in colour-coded images** | | | | | | | |
| --- | --- | --- | --- | --- | --- | --- | --- |
|  | Triplanar view with colour-coding (minimum greyscale intensity I = 0 coded red (RGB, 255 : 0 : 0), greyscale intensity I = 2200 coded white (RGB, 255 : 255 : 255), maximum greyscale intensity I = 4000 coded blue (RGB, 0 : 0 : 255) (Figure 3). | | | | | | |
|  | Validate borders first in coronal view (landmarks as above, Figure 1E-H), then in transverse view (Figure 1I-K), and finally in sagittal (Figure 1L) view. | | | | | | |
|  | - | Preoptic hypothalamus: coded red. | | | | | |
|  | - | Anterior, tuberal, and posterior hypothalamus: coded red and white. | | | | | |
|  | - | Include voxels coded blue only if they belong to the fornix, the pallidohypothalamic fibres, the mamillary body, or the junction of the mamillary body with the mamillary fasciculus. | | | | | |
|  | - | | Preferentially select landmarks in order of hierarchy – as described below (‘>’); in case of doubt select that which can be identified in most of the canonical planes. Landmarks divided by a comma (‘,’) are of the same hierarchical level. | | | | |
|  | Corrections | | | | | | |
|  | - | | Reduce projections and indentions that deviate from the outline of the mask to the maximum length of one voxel. Erase (or fill) single-voxel dots (or holes). | | | | |
|  | - | | Check each correction and reject those that cause implausible changes in any view. This may require revisiting the selection of voxels in step 2. | | | | |
|  | **Transverse landmarks** | | | **Anterior** | **Lateral** | **Medial** | **Posterior** |
|  |  | **Transverse level of anterior commissure** (Figure 1I) | | Anterior commissure | Stria medullaris of thalamus, inferior thalamic peduncle | Midline between hypothalami, third ventricle | Thalamus > mamillo-thalamic tract |
|  |  | **Transverse level of diagonal band** (Figure 1J) | | Diagonal band | Diagonal band, stria medullaris of thalamus, inferior thalamic peduncle, cerebral peduncle, ansa lenticularis, mamillo-tegmental tract | Midline between hypothalami, third ventricle, lamina terminalis | End of MB > posterior margin of mamillo-thalamic tract |
|  |  | **Transverse level of optic tract** (Figure 1K) | | Cerebral exterior | Optic tract, ansa lenticularis | Third ventricle, minimal intersection area* in sagittal plane between MBs | End of MB, cerebral peduncle |
|  | **Sagittal landmarks** | | | **Anterior** | **Superior** | **Inferior** | **Posterior** |
|  |  | **Medial – lateral** (Figure 1L) | | Diagonal band | Hypothalamic sulcus, inferior thalamic peduncle, thalamus | Cerebral exterior | field H2, mamillary fasciculus, zona incerta |
| **4. Finish** | | | | | | | |
|  | Triplanar view of colour-coded images. Check coronal, transverse, and sagittal masks for obvious mistakes. | | | | | | |
|  | Convert back to greyscale images. Zoom in and check boundary voxels between brain matter and cerebrospinal fluid for missing, low-intensity voxels (complete masking, except for non-hypothalamic voxels). | | | | | | |

* include in mask; MB: mamillary body; RGB: red-green-blue colour model. Anatomical nomenclature adopted from the atlas by Mai et al. [23].
